# Adolescents’ perceptions of food outlets in the school neighbourhood and their unhealthy snacking behaviour on the way to and from school

**DOI:** 10.1017/S1368980024001782

**Published:** 2024-10-07

**Authors:** Margaretha Liliana Situmorang, Sandra Mandic, Michael Keall, Melody Smith, Niamh Donnellan, Kirsten J Coppell

**Affiliations:** 1 Department of Medicine, University of Otago, Dunedin, New Zealand; 2 Centre for Sustainability, University of Otago, Dunedin, New Zealand; 3 AGILE Research Ltd., Wellington, New Zealand; 4 Faculty of Health and Environmental Sciences, School of Sport and Recreation, Auckland University of Technology, Auckland, New Zealand; 5 Department of Public Health, University of Otago Wellington, Wellington, New Zealand; 6 School of Nursing, University of Auckland, Auckland, New Zealand; 7 Department of Medicine, University of Otago Wellington, Wellington, New Zealand; 8 Nelson Marlborough Institute of Technology, Nelson, New Zealand; 9 School of Nursing and Midwifery, Edith Cowan University, Joondalup, Western Australia

**Keywords:** Adolescents, Unhealthy snack, School transport modes, School neighbourhood, Food outlets, Neighbourhood deprivation

## Abstract

**Objective:**

To understand the relationship between adolescents’ unhealthy snacking behaviour during their school journey and their perceived and objective measures of food outlet availability in the school neighbourhood.

**Design:**

A cross-sectional survey enquired about socio-demographic information, school transport modes, perceived presence of food outlets in the school neighbourhood and unhealthy food purchase and consumption on the school journey. A geographical information system analysis of the food outlets within 500 m and 1000 m school buffers was undertaken. Data were analysed using generalised linear mixed modelling.

**Setting:**

All twelve secondary schools in Dunedin, Aotearoa New Zealand, March 2020–June 2022.

**Participants:**

Adolescents aged 13–18 years (*n* 725) who reported being familiar with their school neighbourhood.

**Results:**

Perceived availability of food outlets in the school neighbourhood was inversely correlated with distance to the closest food outlet from school and positively correlated with food outlet density within 500 m and 1000 m school buffers. Adolescents’ purchase and consumption of unhealthy snacks and drinks during the school journey were associated with perceived availability of food outlets and with shorter distance to the closest food outlet from school. Mixed transport users, girls and those living in high-deprivation neighbourhoods had higher odds of purchasing and consuming unhealthy snacks and drinks during the school journey than active transport users, boys and those living in low-deprivation neighbourhoods, respectively.

**Conclusions:**

Adolescents perceptions of the food environment and close access to food outlets in the school neighbourhood may influence adolescents’ food purchase and consumption behaviours during the school journey.

Unhealthy dietary habits and low levels of physical activity are risk factors for overweight and obesity^(1)^. Exposure to an obesogenic environment (e.g. a high density of fast-food outlets and a lack of walking and cycling facilities) is associated with an increased risk of obesity in young people^(2)^. While nutrition-related knowledge is important for making healthy food choices, the food environment is also considered to have a significant role in food choice-related behaviour and more so than originally reported^(3)^.

The environmental factors that are associated with adolescents’ food choice^(4)^ include the availability, accessibility and affordability of unhealthy food – that is, soft drinks and snack food high in sugar, fat and calories – along the school route, in the school neighbourhood and within the school environment^(5,6)^, collectively referred to as the ‘school food environment’^(7–9)^. Therefore, while active transport to and from school can increase daily physical activity levels and overall health and well-being in adolescents^(10)^, it may also have an unintended impact on adolescents’ snacking behaviours^(11)^, particularly when unhealthy food outlets are present in the school food environment^(7,8)^. For example, in Scotland, 69 % of adolescents aged 12–16 years who walked or cycled past places selling food or drinks during their school journey sometimes bought food or drinks from these places^(8)^. In Aotearoa New Zealand (New Zealand), many schools have a high number of food outlets, such as convenience stores and takeaway food outlets within walking distance^(12,13)^. Furthermore, the number of fast-food outlets around schools in New Zealand has increased over time, for example, from 1 to 4 outlets between 1966 and 2006 in Christchurch city^(14)^, despite school, parental and community pushback against the opening of new outlets^(15)^. Further, the number of food outlets within walking distance of schools tends to be greater in high-deprivation neighbourhoods compared with low-deprivation neighbourhoods^(16)^ and in urban areas compared with rural areas^(13)^. The availability of food outlets and associated advertising and low-cost unhealthy food options in the school neighbourhood can influence adolescents’ access to unhealthy food options and their food purchasing decisions^(7,8)^.

Previous studies have gathered information about the availability of food outlets in the school neighbourhood either using common straightforward objective measures such as those derived using geographic information systems (GIS)^(13,14)^ or subjective/perceived measures such as questioning adolescents about their perceptions of the presence of food outlets^(7,8)^ but not both perceived and objective measures in a single study. Further, differential relationships with health behaviours have been observed when using either objective or perceived measures of neighbourhood environments^(17)^; however, this has not been examined within the context of adolescents’ school food environments. How adolescents perceive their environment and seeking to understand whether exposures that adolescents are aware of have a direct influence on their health behaviours is important and necessary to fully understand their health risks and benefits^(18)^. As both objective and perceived measures possess inherent strengths and limitations and are not interchangeable^(19)^, their concurrent consideration in studies of environments and health behaviours is important to enhance understanding^(20)^. The aims of this study were to examine whether adolescents’ purchase and consumption of unhealthy snack food and soft drinks during the school journey differed by perceived and objective measures of food outlet availability in the school neighbourhood and investigate the correlation between perceived and objective measures of food outlet availability in the school neighbourhood.

## Materials and methods

### Study setting and participants

Study participants were recruited as part of the Built Environment and Active Transport to School Natural Experiment (BEATS-NE) study in Dunedin city, New Zealand between March 2020 and June 2022^(21)^. The BEATS-NE study was part of the overall BEATS Research Programme which was established in 2013^(22)^. Dunedin is the seventh-largest city by population and the second-largest city by territorial land area in New Zealand^(23)^. Adolescents aged 13–18 years were recruited through their schools using BEATS-NE study research methodology detailed elsewhere^(21)^. Briefly, invited students who were enrolled in all twelve secondary schools in Dunedin received study information 2–4 weeks prior to data collection, and those who agreed to participate (47 %) provided signed consent prior to completing the survey. Parental consent was not required.

### Measures

#### Student survey

Participating adolescents completed an online survey (30–40 min) during a school class supervised by research staff. Most components of the survey had been used previously in the BEATS study and the BEATS Rural study^(21,22,24)^. Survey items used in this analysis included demographic characteristics (age, gender and ethnicity), home address, school transport modes, perceptions of the school neighbourhood food environment and unhealthy snacking behaviours during the school journey.

Adolescents self-reported their transport modes for journeys to and from school using different transport mode options and five response categories (‘*never*’, ‘*rarely*’, ‘*sometimes*’, ‘*most of the time*’, and ‘*all of the time*’). Dominant school transport modes (used ‘*most/all of the time*’) and multi-modal transport were used to classify adolescents into three categories: active transport (‘*on foot*’, ‘*by bike*’, *‘by eBike’,* and/or *‘by eScooter’*), motorised transport (‘*by car (driven by others)*’, ‘*by car (driven by myself)*’, ‘*by school bus*’, and/or ‘*by public transport*’) and mixed transport (‘*by bus and on foot*’, ‘*by car and on foot*’, and/or ‘*other modes or combinations’* of active and motorised transport)^(25)^.

The school neighbourhood was defined as the area within 10 to 15 min’ walk in any direction from schools^(26)^. Using the question *‘How well do you know your school neighbourhood?’*, adolescents rated their familiarity with the school neighbourhood using three response categories: *‘very well’*, *‘somewhat’* and *‘not at all’.* Adolescents who responded *‘not at all’* were not asked questions about their school neighbourhood and therefore were excluded from this analysis (*n* 158; 9 % of the total study sample). The perception of the school neighbourhood food environment was reported by adolescents when they responded to the survey statement *‘There are many places that sell food or drinks (e.g. dairies, supermarkets, or cafés) in my school neighbourhood)’* using a four-level Likert scale of agreement (‘*strongly disagree*’, *‘somewhat disagree’, ‘somewhat agree’,* and ‘*strongly agree*’).

The unhealthy snacking questions were based on the food consumption frequency questions in the Health Behaviour in School-age Children survey^(22,27)^. Although there is no consensus on the definition of snacking^(28)^, the term refers to the consumption of small amounts or portions of food or drinks between regular mealtimes, and healthy and unhealthy snacking behaviours can be differentiated based on food types and the relative percentage of calories that they contribute to daily energy intake^(29)^. Snacking on fruits or vegetables is considered to be a healthy behaviour^(30)^, as it provides an opportunity to fulfil adolescents’ energy and nutrition requirements by including a richer variety of food in the daily diet^(31)^, whereas snacking on foods high in salt, fat or sugar, is considered to be poor nutrition or unhealthy. Unhealthy snack foods and drinks, such as oat bars, chocolates, fruit juices, flavoured milk and soft drinks, are often packaged in an easy-to-go shape, which can be appealing for adolescents^(32)^. In the present study, adolescents were asked about their weekly frequency of buying and consuming: (1) unhealthy snack food like sweets, chips, or ice creams and (2) soft drinks, energy drinks or fruit juice (collectively referred to as ‘soft drinks’ hereafter) on the way to and from school, separately. Adolescents reported their unhealthy snacking behaviour using the root question *‘How often do you usually…?’* with six response categories (‘*never*’, ‘*once a week*’, ‘*twice a week*’, ‘*three times a week*’, ‘*four times a week*’, and ‘*five times a week*’)^(24)^.

#### Derivation of environmental data

Environmental variables (adolescents’ home and school neighbourhood deprivation, school buffers and food outlets in the school neighbourhood) were calculated in ArcGIS version 10·8·1 between July and November 2022.

##### Home neighbourhood and school neighbourhood area-level deprivation

Adolescents’ home and school addresses were geocoded to determine their area-level neighbourhood deprivation according to the New Zealand Index of Deprivation (NZDep). NZDep is an area-based measure of deprivation in New Zealand for the smallest Census geographical reporting area (mesh block) based on nine New Zealand Census variables^(33)^. NZDep is displayed as deciles with Decile 1 representing 10 % of the least deprived areas in New Zealand and Decile 10 being the 10 % most deprived areas. For this analysis, adolescents’ home neighbourhood deprivation and each participating school’s neighbourhood deprivation were regrouped into three categories: low-deprivation (NZDep 1–3), mid-deprivation (NZDep 4–7) and high-deprivation (NZDep 8–10).

##### School neighbourhood buffers

The school neighbourhood was defined using two street network buffers of 500 m and 1000 m distances around each school^(21)^. There is no consensus on the optimal buffer distance for defining neighbourhood in studies of adolescents^(34)^. Ideally, multiple buffers should be used, and there is some evidence that larger buffers (i.e. >800 m) might be the most appropriate for describing adolescents’ neighbourhoods^(34)^. The buffers used for the overall BEATS-NE study were based on previous research including one study in New Zealand that reported 46·8 % of urban schools had a convenience store within 500 m from the school and the median of the closest distance to a convenience store from schools was 535 m (interquartile range: 291–929 m)^(13)^. Therefore, capturing the school neighbourhood using two buffer zones of 500 m and 1000 m was deemed the most appropriate approach for this study.

##### Food outlets

All food outlets within the Dunedin City Council boundary were identified. Food outlet data were generated using ‘place types’, which is a function of the Google Maps Platform that supports place searches in Google Places Application Programming Interface, within the Dunedin City Council boundary. The retrieval of food outlet data used two approaches: (1) the selection of food outlet categories from a pre-determined list of 96 ‘*place types*’ and (2) a free text search where Google matches specified text against the places’ name, description and reviews. The Google list of food outlets in Dunedin was audited using a modified virtual ground-truth method^(35)^, where the validity (i.e. presence) of each food outlet was confirmed by two authors (MLS and KC) using Google Maps, Google Street View, food outlets’ webpage or Facebook page, phone calls, local knowledge or visits to food outlet addresses when necessary. All valid food outlets were categorised into one of eight food outlet types (bakery, café, convenience store, fast-food outlet, fresh food store, restaurant, supermarket and takeaway) for this analysis. The definitions of each food outlet type are presented in Supplemental Table 1 (see online supplementary material). Two variables of food outlets in the school neighbourhood were derived for this analysis in ArcGIS: (1) for each school, the distance from the school to the closest food outlet was calculated using a custom geoprocessing script using the origin-destination tool and (2) density (total food outlet counts per kilometre square area) within the 500 m and 1000 m school buffers was derived using the intersect tool.

### Data analysis

Demographic characteristics were analysed using descriptive statistics. Continuous data were reported as mean ± sd and categorical data as frequency (%). A generalised linear mixed model was used to examine the association between adolescents’ unhealthy snacking behaviour and the availability of food outlets in their school neighbourhood. School variable was included as a random effect to account for the clustering of adolescents within schools. The outcome variable for unhealthy snacking behaviour was a dichotomous categorical variable (never *v*. purchased and consumed unhealthy snack food or soft drinks during the school journey ≥1 per week). Both bivariable and multivariable models used adolescents’ perceptions of the presence of food outlets in their school neighbourhood, distance to the closest food outlet from school and density of all food outlets within the 500 m and 1000 m buffers to categorise the school neighbourhood food environment. A Pearson product-moment correlation was used to check the multicollinearity between the food environment variables, and a correlation coefficient of *r* < 0·80 was assumed to indicate that collinearity between variables was not a concern. To examine associations with the food outlet density variable (a continuous variable), adolescents were categorised into three groups based on having an approximately equal number of participants within each food outlet density range. The multivariable models included all food outlet variables used in the bivariable model with individual-level factors as covariates (age, gender, school transport mode and home neighbourhood deprivation). Adolescents’ age groups were categorised into two groups (below 16 years and ≥16 years) because 16 is the age of eligibility for a person to obtain a restricted driving license in New Zealand^(36)^. A *P* < 0·05 was considered statistically significant. Data were analysed using SPSS software (version 27·0, IBM, Armonk, NY, USA).

## Results

Of the 1795 consenting adolescents with valid survey data, 1070 were excluded from this analysis for the following reasons: boarding at school or privately (*n* 234), missing unhealthy snacking data (*n* 678) and reported not being familiar with the school neighbourhood (*n* 158). Among the 725 adolescents included in this analysis, the mean age was 15·2 ± 1·4 years, 52·4 % were girls, 65·8 % were New Zealand European and 59·0 % lived in a low-deprivation neighbourhood area (Table 1). The median distance from home to school was 3·9 km, and about one-fifth of the adolescents used active transport on the way to (20·3 %) or from (22·4 %) school. There were three schools located in a low-deprivation area, three in a medium-deprivation area and six in a high-deprivation area.

**Table 1:** Socio-demographic characteristics and school travel modes of study participants (*n* 725)

	Groups	Participants *n* (%) unless otherwise specified	
		Mean or *n*	sd or %
Age (mean ± sd; years)		15·2	1·4
Gender	Male	328	45·2 %
	Female	380	52·4 %
	Gender diverse	17	2·3 %
Ethnicity	New Zealand European	477	65·8 %
	Māori	104	14·3 %
	Pacific Islands	20	2·8 %
	Asian	34	4·7 %
	Other	90	12·4 %
Distance to school* (median (IQR); km)	Walkable network	3·9	1·8–8·4
Home neighbourhood deprivation†	Low (NZDep deciles 1–3)	348	48·3 %
	Mid (NZDep deciles 4–7)	241	33·5 %
	High (NZDep deciles 8–10)	131	18·2 %
School neighbourhood deprivation†	Low (NZDep deciles 1–3)	212	29·2 %
	Mid (NZDep deciles 4–7)	179	24·7 %
	High (NZDep deciles 8–10)	334	46·1 %
Transport to school‡	Active transport	143	20·3 %
	Motorised transport	376	53·4 %
	Mixed transport	185	26·3 %
Transport from school§	Active transport	155	22·4 %
	Motorised transport	342	49·5 %
	Mixed transport	194	28·1 %

* Distance to school data were missing for twenty-nine adolescents.

† Home and school neighbourhood deprivation data were categorised using the NZDep deciles then recategorised into three groups. There were missing home neighbourhood deprivation data for five adolescents.

‡ Transport to school data were missing for twenty-one adolescents.

§ Transport from school data was missing for thirty-four adolescents. IQR: interquartile range; sd: standard deviation.

### Food outlets in the school neighbourhood

The average distance from each of the twelve schools to the closest food outlet was 0·49 km (range: 0·18–0·87 km). The total food outlet counts within the 500-m school buffer varied from no food outlets for six schools to forty-five food outlets for one school, and the mean density of food outlets was 13·5 outlets/km^2^. For the 1000-m school buffer, the total food outlet count ranged from two for one school to 153 food outlets for one school, with more than 100 food outlets for four schools. The mean density of food outlets within the 1000-m buffer was 29·9 outlets/km^2^. Restaurants were the most common type of food outlet within both the 500-m and 1000-m school buffers. The six schools in high-deprivation areas had the highest density of food outlets within the 500 m buffer, and the three schools in mid-deprivation areas had the highest density of food outlets within the 1000-m buffer (see online supplementary material, Supplemental Table 2). Cafés and fast-food outlets were found within the 500-m buffer of schools in high-deprivation areas only.

### Perceived and objective measures of the food environment in the school neighbourhood

Adolescents’ perceptions of the school neighbourhood food environment were weakly correlated with objective measures of food outlet density within the 500-m and 1000-m school neighbourhood buffers (Table 2). The distance from school to the closest food outlet was negatively correlated with adolescent’s perceptions that there were many food outlets (perceived presence of food outlets) in their school neighbourhood. Within the 500-m school neighbourhood buffer, the perceived presence of food outlets in the school neighbourhood was positively correlated with the density of bakeries and all food outlet types. For the 1000-m school neighbourhood buffer, the perceived presence of food outlets was positively correlated with the density of bakeries, fresh food stores and supermarkets.

**Table 2:** The correlation between GIS-measured food outlets and perceived presence of food outlets in the school neighbourhood

		Perceived presence of food outlets	
		*r*	*P-value*
Distance to the closest food outlets from school		–0·08	**0**·**031**
Food outlets density (n/km^2^) within 500 m buffer	Bakery	0·16	**<0**·**001**
	Café	0·06	0·088
	Convenience store	0·02	0·526
	Fast-food	0·04	0·350
	Fresh food store	*N/A*	*N/A*
	Restaurant	0·07	0·050
	Supermarket	*N/A*	*N/A*
	Takeaway	0·05	0·187
	All food outlet types	0·07	**0·048**
Food outlets density (n/km^2^) within 1000 m buffer	Bakery	0·09	**0·014**
	Café	–0·02	0·590
	Convenience store	–0·03	0·420
	Fast-food	–0·03	0·474
	Fresh food store	0·07	**0·046**
	Restaurant	–0·03	0·406
	Supermarket	0·10	**0·005**
	Takeaway	0·00	0·952
	All food outlet types	–0·02	0·604

GIS, geographical information system.

N/A: Data are not available since this food outlet type did not exist within 500 m buffer.

### School neighbourhood food environment and adolescents’ unhealthy snacking during the school journey

Overall, 22·8 % of adolescents purchased and consumed unhealthy snack food or soft drinks on the way to school, and 43·0 % on the way from school at least 1 day per school week. The proportion of adolescents who purchased and consumed unhealthy snack food or soft drinks ≥1 d per week during the school journey differed by their transport modes on the way to school (active/motorised/mixed: 21·0 %/18·4 %/31·4 %; *P* = 0·002) and from school (active/motorised/mixed: 38·4 %/42·4 %/51·5 %; *P* < 0·001). Table 3 presents the odds for adolescents’ purchase and consumption of unhealthy snack food or soft drinks (‘≥1 per week’ *v*. ‘never’) on the way to and from school by objective measures of food outlets and adolescents’ perceptions of the food outlets in their school neighbourhood. Adolescents attending schools with the closest food outlets located beyond 500 m from school had lower odds of unhealthy snacking during the school journey than those who attended schools with the closest food outlets within 500 m of the school. A lower OR of unhealthy snacking on the way to school, but not on the way from school, was also found among adolescents who perceived the availability of food outlets in their school neighbourhood than those who did not. There was no significant difference in the OR of unhealthy snacking on the way to and from school by the density of food outlets within the 500-m and 1000-m school buffer.

**Table 3:** Crude OR of adolescents’ unhealthy snacking on the way to and from school

	Groups	Purchased and consumed unhealthy snack food or soft drinks					
		On the way to school (*n 725*)			On the way from school (*n 725*)		
		OR	95 % CI	*P-value*	OR	95 % CI	*P-value*
Objective measures of food outlets							
Distance to the closest food outlets from school	Within 500 m (ref)	1·00			1·00		
	Beyond 500 m and within 1000 m	0·69	0·48, 0·99	**0·046**	0·71	0·53, 0·96	**0·027**
Density (n/km^2^) of all food outlets within 500 m buffer*	None (ref)	1·00			1·00		
	>0 and ≤5	1·22	0·83, 1·79	0·312	1·37	0·99, 1·91	0·058
	5·01 or more	1·01	0·61, 1·68	0·968	1·16	0·76, 1·77	0·495
Density (n/km^2^) of all food outlets within 1000 m buffer*	<4 (ref)	1·00			1·00		
	4 to 12	0·91	0·59, 1·39	0·654	1·04	0·73, 1·49	0·833
	12·01 or more	1·09	0·70, 1·71	0·702	1·15	0·78, 1·68	0·482
Perceived food environment							
Food outlets†	Disagree (ref)	1·00			1·00		
	Agree	0·57	0·39, 0·84	**0·004**	0·99·	0·70, 1·40	0·964

* For the variable of food outlet density, adolescents were categorised into three groups based on having an approximately equal sample size within each group.

† Perceived presence of food outlets defined by whether adolescents disagreed (*‘somewhat/strongly disagree*’) or agreed (*‘somewhat/strongly agree*’) that there were many food outlets in their school neighbourhood.

The adjusted ORs of adolescents’ unhealthy snacking on the way to and from school in relation to food outlet variables in the school neighbourhood controlling for individual factors are presented in Table 4. A lower OR of unhealthy snacking on the way to school was found for adolescents attending schools where the distance to the closest food outlet was beyond 500 m but within 1000 m from school, and among adolescents who perceived availability of food outlets in their school neighbourhood than those who did not. In contrast, a higher OR of unhealthy snacking on the way to school was found among adolescents who resided in high-deprivation neighbourhoods compared to those who resided in low-deprivation neighbourhoods and among mixed transport users compared to those who used active transport (walking, cycling or scootering) to school. On the way from school, higher OR of unhealthy snacking was also found among mixed transport users than those who used active transport. Higher OR of unhealthy snacking on the way from school was also found in girls compared to boys and adolescents who lived in mid-deprivation compared to high-deprivation neighbourhoods. There were no significant associations for unhealthy snacking during the school journey with adolescents’ age or with the density of food outlets within the 500-m or 1000-m buffers.

**Table 4:** OR of adolescents purchasing and consuming unhealthy snacks on the way to and from school adjusted for distance, density and perceived presence of food outlets in the school neighbourhood and individual covariates

		Purchased and consumed unhealthy snack food or soft drink					
	Groups	On the way to school *(n 700)*			On the way from school *(n 686)*		
		Adjusted OR§	95 % CI	*P–value*	Adjusted OR§	95 % CI	*P–value*
Objective measures of food outlets							
Distance to the closest food outlets from school	Within 500 m *(ref)*	1·00			1·00		
	Within 1000 m but beyond 500 m	0·35	0·16, 0·75	**0·007**	0·86	0·44, 1·67	0·647
Density (n/km^2^) of all food outlets within 500 m buffer	None *(ref)*	1·00			1·00		
	>0 and ≤5	0·57	0·23, 1·40	0·219	1·08	0·50, 2·37	0·840
	5·01 or more	0·47	0·18, 1·23	0·124	1·03	0·45, 2·38	0·938
Density (n/km^2^) of all food outlets within 1000 m buffer	<4 *(ref)*	1·00			1·00		
	4 to 12	1·10	0·57, 2·16	0·771	1·12	0·65, 1·94	0·682
	12·01 or more	1·08	0·56, 2·08	0·817	1·13	0·66, 1·91	0·656
Perceived food environment							
Food outlets*	Disagree *(ref)*	1·00			1·00		
	Agree	0·64	0·42, 0·99	**0·042**	0·96	0·66, 1·39	0·120
Individual-level covariates							
Age	<16 years *(ref)*	1·00			1·00		
	16 years or more	1·04	0·66, 1·65	0·869	1·04	0·71, 1·53	0·846
Gender	Boys *(ref)*	1·00			1·00		
	Girls	0·80	0·51, 1·25	0·325	1·53	1·05, 2·22	**0·026**
	Gender diverse	1·29·	0·42, 3·98	0·657	1·33·	0·47, 3·80	0·589
School transport modes†	Active transport *(ref)*	1·00			1·00		
	Motorised transport	0·88	0·53, 1·45	0·614	1·30	0·86, 1·97	0·214
	Mixed transport	1·78·	1·04, 3·04	**0·035**	1·99·	1·26, 3·16	**0·003**
Home neighbourhood deprivation‡	Low *(ref)*	1·00			1·00		
	Mid	1·26	0·82, 1·94	0·290	1·70	1·19, 2·42	**0·004**
	High	1·82	1·11, 2·98	**0·019**	1·51	0·98, 2·35	0·064

* Perceived presence of food outlets defined by whether adolescents disagreed (*‘somewhat/strongly disagree*’) or agreed (*‘somewhat/strongly agree*’) that there were many food outlets in their school neighbourhood.

† Transport modes to school data were not available for twenty-one participants (3 % of the total sample). Transport modes from school data were not available for thirty-four participants (5 % of the total sample).

‡ Home neighbourhood deprivation data were not available for five participants (1 % of the total sample).

§ The multivariable regression fitted school as a random effect.

## Discussion

This study examined the association between adolescents’ perceptions, and objective measures, of food outlet availability in their school neighbourhood and adolescents’ unhealthy snacking during their school journey among those who reported being familiar or somewhat familiar with their school neighbourhood in Dunedin city, New Zealand. The key findings were: (1) The average distance from secondary schools in Dunedin city to food outlets was 0·49 km, and all twelve schools in the city had two or more food outlets within 1000 m; (2) Adolescents’ perception of food outlet availability in their school neighbourhood was negatively correlated with the distance from their school to the closest food outlet and the density of food outlets within a 500-m school buffer; (3) Proximity to, and adolescents’ perceived presence of, food outlets in the school neighbourhood were associated with the purchase and consumption of unhealthy snacks and drinks on the way to school, but not from school and (4) Gender, school transport mode and home neighbourhood deprivation were significant individual covariates associated with adolescents’ purchase and consumption of unhealthy snacks and drinks during the school journey. Taken together, these findings suggest that adolescents’ purchase and consumption of unhealthy snacks and drinks during the school journey was associated with objective and perceived measures of food outlets in their school neighbourhood and was also linked to their gender, school transport modes and home neighbourhood deprivation.

In this Dunedin city, New Zealand, study, the density of food outlets within the 500-m and 1000-m school buffer varied, which may justify the use of multiple buffer analyses to better capture the overall food environment in the school neighbourhood^(34)^. The density of food outlets was higher for schools in the mid- and high-deprivation areas compared with low-deprivation neighbourhood areas. This observation is consistent with other studies that have also demonstrated a relationship between food outlet availability and neighbourhood deprivation^(16,37)^. The significant association between adolescents’ unhealthy snacking and objective measures of the school neighbourhood food environment was found for the proximity, but not the density, of food outlets. Several previous studies have also found inverse associations between unhealthy food purchases and proximity to food outlets in the school neighbourhood^(38,39)^. This suggests that a shorter distance to food outlets makes it unsurprisingly easier for adolescents to purchase and consume unhealthy food and drinks. In contrast to other studies^(40,41)^, we found no significant association between food outlet density in the school neighbourhood and unhealthy snacking, which may be attributed to the fact that six of twelve schools did not have any food outlets within the 500-m buffer. Another potential explanation is the inclusion of all types of food outlets in this study, in contrast to previous research where specific types of food outlets (e.g. convenience store or fast-food outlets) have been the main focus^(7,16)^. Further, unlike many other studies, the binary approach of classifying food outlets as either healthy or unhealthy was not used in this study since unhealthy snacks can be purchased from food outlets that are ostensibly considered healthy yet concurrently sell and promote unhealthy food (e.g. supermarkets)^(42)^.

In this study, adolescents’ perceived the availability of food outlets in their school neighbourhood was negatively correlated with the distance to the closest food outlet from their school. This observation can be explained by considering the positive relationship between distance and the time it takes to reach food outlets. When the distance to food outlets is shorter, there is a stronger consensus regarding their perceived availability^(19)^. Adolescents who agreed that there were many food outlets available in their school neighbourhood had a lower OR of unhealthy snacking than those who disagreed. There could be several reasons for this unexpected association. Firstly, perceiving a high availability of food outlets may provide adolescents with greater access to a greater variety of food and drink choices, making it easier for them to choose healthy options^(43)^. Secondly, the perception of a greater number of food outlets may be associated with a higher level of awareness and consciousness among adolescents about the surrounding food environment and healthy eating, which is related to healthy food and drink choices^(44)^. However, there may be other possible explanations which would require further investigation to fully explore these potential relationships.

Objective and perceived measures of the food environment in the school neighbourhood were weakly correlated in this study, and different relationships with unhealthy snacking were found for both measures. These results suggest that objective measures and adolescents’ perceptions of food outlet availability may not be directly related and may be associated differently with the purchasing and consumption of food and drinks sourced from the neighbourhood^(40,43)^. Specifically, this implies that relying solely on either objective measures or perceptions to assess the food environment may not provide a complete picture of the food environment and its potential impact on adolescents’ snacking behaviours. Therefore, assessing both physical access to food outlets and subjective perceptions of such access is considered essential in order to better understand unhealthy food exposures^(20)^.

A higher OR of unhealthy snacking was found in girls, mixed transport users and those residing in mid- and high-deprivation neighbourhoods compared with boys, active transport users and those residing in low-deprivation neighbourhoods. It seems possible that girls are more susceptible to their surrounding food environment, including advertising, than boys, a factor that could potentially facilitate their engagement in unhealthy snacking behaviours^(45)^. Adolescents using mixed transport may live further from their school than active transport users, in which the longer distances or longer school trip durations may have delayed or restricted access to food, thus increasing the likelihood of hunger during the between home and school period and prompting a stop for food during the school journey^(46)^. Furthermore, the significant association between unhealthy snacking and home neighbourhood deprivation may be explained by the high availability of food outlets and food insecurity among adolescents living in areas of higher deprivation^(37,47)^.

### Implications

This study emphasises the importance of using both objective and perceived measures together to provide a comprehensive view of food environments and the potential role of the food environment around schools in shaping adolescents’ unhealthy snacking behaviours. In particular, the distance to food outlets was directly correlated with how adolescents perceived the availability of food outlets in their school neighbourhood and was associated with their unhealthy snacking during the school journey. While schools in New Zealand have been encouraged to create their own healthy food policy based on Ministry of Health guidance for healthy food and drink provision^(48)^, it is necessary to not only limit access to unhealthy food inside schools but also outside schools to promote healthy eating behaviours in adolescents. Even if the in-school food environment provides healthy food choices for students, having access to unhealthy food from local food outlets in the school neighbourhood may pose a barrier to encouraging healthy eating behaviours^(7,49)^. Exposure to many unhealthy foods may blur adolescents’ understanding and preference of healthy and unhealthy foods^(7,50)^, and if the blurred understanding and high availability of unhealthy foods are commonplace, adolescents may face food choice fatigue and may make their choices based on accessibility in the everyday environment^(7,50)^.

### Study strengths and limitations

The strengths of this study are the participation of all twelve secondary schools in Dunedin city, the large number of participants (*n* 725), the inclusion of both perceived and objective measures of the school neighbourhood food environment, the inclusion of two different school neighbourhood buffers (500 m and 1000 m) and the assessment of unhealthy snack food and soft drink purchases and consumption on the journeys both to school and from school. This research also has limitations. Unhealthy snacking behaviour was self-reported, which may have introduced responder bias due to social desirability. However, the survey questions were carefully worded to help minimise bias; for example, the use of healthy or unhealthy was not used to describe the type of food or drinks. The food environment data relied on a Google Places Application Programming Interface retrieved list and was limited to the retrieval of places from a predetermined list, and food outlets with a different description may not have been included (e.g. dairy). The implication is that not all food outlets may have been identified and included in this analysis. However, the research team had good local knowledge of food outlets in the area studied and the modified virtual ground-truth method used meant the number of food outlets missed or misclassified was likely to have been very small. Generalisability is limited to the study city context, where the socio-demographic characteristics of participants were largely representative of the area (but were less ethnically diverse than for the New Zealand adolescent population), although participants excluded from the analysis were slightly younger and there were slightly higher proportions of boys and New Zealand Europeans compared with those included in the analysis. In addition to socio-demographic differences, varying geographical characteristics such as urbanicity, topography and climate will influence generalisability to other locations. The categorisation and definition of food outlet types used in this study may also differ from those in other studies^(7,16)^ and therefore the results of this study may not be directly comparable.

Future research on adolescents’ unhealthy snacking behaviour during the school journey and its association with the school neighbourhood food environment should also consider factors such as food advertising, food discounts, special promotions of unhealthy food and drinks and targeted sales to young adolescent populations. Additionally, studying the food environments of other neighbourhood spaces where adolescents gather or spend time after school, such as home neighbourhoods or sport and recreation centres, would provide insights into the social influence of the food environment.

In conclusion, the present study suggests that the perceived presence of food outlets and the distance to food outlets in the school neighbourhood were associated with adolescents’ unhealthy snacking behaviour on the way to school but not from school. School transport modes and home neighbourhood deprivation also had a significant association with adolescents’ unhealthy snacking both on the way to and from school. To promote healthy eating among adolescents, policymakers could consider intervention strategies such as zoning regulations for food outlets, limiting unhealthy food sales and promotion and implementing healthy food and drinks guidance within the school neighbourhoods.

## Supporting information

Situmorang et al. supplementary material 1 Situmorang et al. supplementary material

Situmorang et al. supplementary material 2 Situmorang et al. supplementary material

## Supplementary material

For supplementary material accompanying this paper visit https://doi.org/10.1017/S1368980024001782
