# Supplementary material for: Adolescents’ perceptions of food outlets in the school neighbourhood and their unhealthy snacking behaviour on the way to and from school
Source: Public Health Nutr. 2024 Oct 7;27(1):e198. doi: 10.1017/S1368980024001782 (PMC11505387; doi:10.1017/S1368980024001782)
Supplement: Situmorang et al. supplementary material 1 — Situmorang et al. supplementary material [file S1368980024001782sup001.docx]

# Supplemental Table 1 *The definitions of the eight different food outlet types used in the GIS analysis*

| Food outlet classification | Definition | |
| --- | --- | --- |
| Bakery | | A food retail store characterised by baked goods made on site, with products predominantly for meal preparation at home (e.g., loaf of bread) and for special occasions (birthday cake, wedding cake). Minimal sales on ready-to-consume food and beverages. |
| Café | | A food outlet characterised by sales of coffee, tea, pastries, baked goods, and simple meals. Usually, a casual and relaxed atmosphere of dine-in experience which can be suitable for work or socialising. May also provide food sales over the counter for takeaway. |
| Convenience Store | | A food retail store that predominantly sells processed and pre-packaged food and beverages on a smaller scale than a supermarket and with only over the counter check-out service. Fresh product sales of fruit and vegetables are either absent or minimal. This category includes petrol station stores, small grocery stores, confectionary (ice-cream stores), and dairy (which is a food outlet type in the NZ context). |
| Fast-food | | A food outlet that provides quick food service and has some food on display ready for purchase. Characterised by check-out service over the counter, standardized ready-to-eat meals made of processed food, and served in disposable packaging (plastic container, paper food trays). Spaces for dine-in are either absent or minimal. |
| Fresh food stores | | A food retail store that predominantly sells fresh and perishable products such as fruit and vegetables, fish, and meat. Might also sell raw ingredients (seeds, spices, and beans). No or minimal sales of packaged and processed ready-to-eat food products. |
| Restaurants | | A food outlet with dine-in experience with tables and wait staff. A more formal atmosphere compared to a café with food prepared by order. |
| Supermarkets | | A food retail store characterised by a large variety of food products including fresh products (fruit, vegetables, fish, meat), raw ingredients for meal preparation at home, and ready-to-eat food and beverages. Typically, long opening hours and may provide over the counter service as well as self-checkout. |
| Takeaway | | A food-service provider that makes food by order for consumption elsewhere. Characterised by quick service (fish and chips, Asian/Indian takeaways, pizza), served in disposable packaging, and order and payment over counter. Spaces for dine-in are either absent or minimal. |
