# Supplementary material for: Adolescents’ perceptions of food outlets in the school neighbourhood and their unhealthy snacking behaviour on the way to and from school
Source: Public Health Nutr. 2024 Oct 7;27(1):e198. doi: 10.1017/S1368980024001782 (PMC11505387; doi:10.1017/S1368980024001782)
Supplement: Situmorang et al. supplementary material 2 — Situmorang et al. supplementary material [file S1368980024001782sup002.docx]

**Supplemental Table 2** *The mean counts and density of different food outlet types within 1000 m buffer for schools located in low-, mid-, and high-deprivation neighbourhood areas^1^*

| **Food outlet types** | **Food outlet density (n/km^2^) within 500 m school buffer** | | | **Food outlet density (n/km^2^) within 1000 m school buffer** | | |
| --- | --- | --- | --- | --- | --- | --- |
|  | **By school neighbourhood deprivation** | | | **By school neighbourhood deprivation** | | |
|  | **Low** *(N = 3)* | **Mid** *(N = 3)* | **High** *(N = 6)* | **Low** *(N = 3)* | **Mid** *(N = 3)* | **High** *(N = 6)* |
|  | **Mean**  **(Min – Max)** | **Mean**  **(Min – Max)** | **Mean**  **(Min – Max)** | **Mean**  **(Min – Max)** | **Mean**  **(Min – Max)** | **Mean**  **(Min – Max)** |
| **Bakery** | 0.72 (0.00 – 2.16) | 0.00 (0.00 – 0.00) | 0.00 (0.00 – 0.00) | 0.95 (0.80 – 1.09 | 0.52 (0.00 – 1.03) | 0.37 (0.00 – 1.13) |
| **Café** | 0.00 (0.00 – 0.00) | 0.00 (0.00 – 0.00) | 5.01 (0.00 – 23.11) | 0.36 (0.00 – 1.09) | 9.88 (0.00 – 15.92) | 8.17 (0.56 – 22.52) |
| **Convenience Store** | 0.00 (0.00 – 0.00) | 0.81 (0.00 – 2.44) | 1.35 (0.00 – 4.62) | 0.00 (0.00 – 0.00) | 1.77 (1.15 – 2.11) | 1.93 (0.00 – 5.28) |
| **Fast-Food** | 0.00 (0.00 – 0.00) | 0.00 (0.00 – 0.00) | 1.54 (0.00 – 6.93) | 0.36 (0.00 – 1.09) | 6.07 (0.00 – 9.25) | 3.88 (0.00 – 12.33) |
| **Fresh Food Store** | N/A | N/A | N/A | 0.00 (0.00 – 0.00) | 0.00 (0.00 – 0.00) | 0.50 (0.00 – 1.08) |
| **Restaurant** | 3.58 (0.00 – 8.59) | 0.70 (0.00 – 2.10) | 13.29 (0.00 – 69.34) | 2.25 (0.00 – 4.36) | 23.33 (1.73 – 37.97) | 13.01 (0.00 – 40.54) |
| **Supermarket** | N/A | N/A | N/A | 0.36 (0.00 – 1.09) | 0.51 (0.00 – 1.54) | 1.53 (0.00 – 3.38) |
| **Takeaway** | 0.95 (0.00 – 2.86) | 0.00 (0.00 – 0.00) | 2.50 (0.00 – 10.40) | 1.81 (0.80 – 2.73) | 4.41 (1.73 – 7.38) | 4.01 (0.00 – 9.40) |
| **All food outlets** | 5.26 (0.00 – 11.45) | 1.51 (0.00 – 2.44) | 23.69 (0.00 – 104.01) | 6.10 (2.85 – 11.45) | 46.49 (4.60 – 70.66) | 33.40 (2.67 – 86.15) |

^1^ School neighbourhood deprivation data were categorised using the NZDep deciles then recategorised into three groups (Low: NZDep 1–3; Mid: NZDep 4–7; High: NZDep 8–10); N/A: Data are not available since this food outlet type did not exist within 500 m buffer
